# Impulsivity is associated with prospective amyloid PET accumulation and cognitive decline in cognitively normal older adults

**DOI:** 10.1002/alz70861_108478

**Published:** 2025-12-23

**Authors:** Tyler N Toueg, Michael A Silver, William J. Jagust

**Affiliations:** ^1^ University of California, Berkeley, Berkeley, CA USA

## Abstract

**Background:**

Behavior and personality are associated with risk for Alzheimer’s disease (AD). Impulsivity shares some features with known risk factors but has never been evaluated in cognitively normal older adults (CN) at risk for AD.

**Methods:**

We investigated relationships between AD PET biomarkers (PiB PET and FTP PET), impulsivity (Barratt Impulsiveness Scale, BIS) and cognition (episodic memory composite, non‐memory composite, and Mini Mental State Examination) in 149 CN. Linear regression was used to assess the association between PET biomarkers and BIS total score. LASSO regression was used to identify the BIS factors most associated with PET biomarkers. Individual subject slopes for PET, impulsivity, and cognition were estimated using linear mixed effects models with a fixed effect of time elapsed and random effects for each participant’s slope and intercept.

**Results:**

All PET biomarkers were cross‐sectionally associated with BIS total score, with the strongest association observed for the BIS factor Cognitive Instability (CI). Baseline CI was associated with longitudinal global amyloid PET slope. Baseline CI was also associated with prospective decline in non‐memory cognition. In this relationship, baseline CI interacted with amyloid status, such that baseline CI was only associated with prospective cognitive decline in amyloid‐positive subjects.

**Conclusion:**

Our findings suggest that impulsivity, especially cognitive instability, may increase risk for AD and cognitive decline in CN.

